# Modulated Response of *Aspergillus fumigatus* and *Stenotrophomonas maltophilia* to Antimicrobial Agents in Polymicrobial Biofilm

**DOI:** 10.3389/fcimb.2020.574028

**Published:** 2020-10-06

**Authors:** Lolita Roisin, Elise Melloul, Paul-Louis Woerther, Guilhem Royer, Jean-Winoc Decousser, Jacques Guillot, Eric Dannaoui, Françoise Botterel

**Affiliations:** ^1^EA 7380 Dynamyc, Université Paris-Est Créteil, Ecole nationale vétérinaire d'Alfort, USC Anses, Créteil, France; ^2^Unité de Bactériologie-Hygiène, Département de prévention, diagnostic et traitement des infections, Hôpital Henri Mondor, AP-HP, Créteil, France; ^3^LABGeM, Génomique Métabolique, CEA, Genoscope, Institut François Jacob, Université d'Evry, Université Paris-Saclay, CNRS, Evry, France; ^4^Unité de Parasitologie-Mycologie, Ecole nationale vétérinaire d'Alfort, Maisons-Alfort, France; ^5^Unité de Parasitologie-Mycologie, Service de Microbiologie, Hôpital Européen Georges Pompidou, AP-HP, Université Paris-Descartes, Paris, France; ^6^Unité de Parasitologie-Mycologie, Département de prévention, diagnostic et traitement des infections, Hôpital Henri Mondor, AP-HP, Créteil, France

**Keywords:** *Aspergillus fumigatus*, *Stenotrophomonas maltophilia*, polymicrobial biofilm, antimicrobial susceptibility, antifungal agent, antibacterial agent

## Abstract

**Introduction:** The complexity of biofilms constitutes a therapeutic challenge and the antimicrobial susceptibility of fungal-bacterial biofilms remains poorly studied. The filamentous fungus *Aspergillus fumigatus* (Af) and the Gram-negative bacillus *Stenotrophomonas maltophilia* (Sm) can form biofilms and can be co-isolated from the airways of cystic fibrosis (CF) patients. We previously developed an *in vitro* biofilm model which highlighted the antibiosis effect of Sm on Af, which was dependent on the bacterial fitness. The aim of the present study was to investigate the *in vitro* susceptibility of Af and Sm in mono- or polymicrobial biofilms to five antimicrobial agents alone and in two-drug combinations.

**Methods:** Af and Sm clinical reference strains and two strains from CF sputa were tested through a planktonic and biofilm approaches. Af, Sm, or Af-Sm susceptibilities to amphotericin B (AMB), itraconazole (ITC), voriconazole (VRC), levofloxacin (LVX), and rifampicin (RFN) were evaluated by conventional planktonic techniques, crystal violet, XTT, qPCR, and viable plate count.

**Results:** Af planktonic cells and biofilms in formation were more susceptible to AMB, ITC, and VRC than Af mature biofilms. Af mature biofilms were susceptible to AMB, but not to ITC and VRC. Based on viable plate count, a lower concentration of LVX and RFN was required to reduce Sm cell numbers on biofilms in formation compared with mature biofilms. The antibiosis effect of Sm on Af growth was more pronounced for the association of CF strains that exhibited a higher fitness than the reference strains. In Af-Sm biofilms, the fungal susceptibility to AMB was increased compared with Af biofilms. In contrast, the bacterial susceptibility to LVX decreased in Af-Sm biofilms and was fungal biomass-dependent. The combination of AMB (64 μg/mL) with LVX or RFN (4 μg/mL) was efficient to impair Af and Sm growth in the polymicrobial biofilm.

**Conclusion:** Sm increased the Af susceptibility to AMB, whereas Af protected Sm from LVX. Interactions between Af and Sm within biofilms modulate susceptibility to antimicrobial agents, opening the way to new antimicrobial strategies in CF patients.

## Introduction

Biofilm-embedded cells have specific characteristics, which distinguish them from planktonic cells, namely their lower susceptibility to drugs explained in particular by the presence of self-produced protective matrix and the reduction of microbial metabolic activity in the biofilm (Desai et al., 2014; Lebeaux et al., 2014; Flemming et al., 2016).

Many microorganisms are commonly co-isolated from the airways of cystic fibrosis (CF) patients (Botterel et al., 2018; Granchelli et al., 2018), including the filamentous fungus *Aspergillus fumigatus* (Burgel et al., 2016) and the Gram-negative bacillus *Stenotrophomonas maltophilia* (Esposito et al., 2017). Approximatively, 10% of the French CF patients carry in their airways *S. maltophilia*, and 30 % carry *Aspergillus* (French, 2019), with common co-infections, as recently shown in a very large cohort of CF patients (Granchelli et al., 2018). Treatment of these pathogens in chronic respiratory diseases is often difficult due to their multidrug-resistant nature, especially for *S. maltophilia*, and to their biofilm-forming ability (Flores-Treviño et al., 2019). The presence of microbial aggregates and biofilms have already been observed in the respiratory tract of CF patients, which is a favorable environment for biofilm formation (Bjarnsholt et al., 2009; Ramage et al., 2011; Kragh et al., 2014). *S. maltophilia* biofilm was documented in the sputum of CF patients (Høiby et al., 2017), although there is no direct evidence supporting the presence of *Aspergillus* biofilm *in vivo* in CF patients.

The complexity of biofilm structure constitutes a therapeutic challenge since infections are often treated with drugs selected according to the results of susceptibility testing of microorganisms in planktonic form. Furthermore, Keays et al. (2009) showed a better clinical outcome when CF patients were treated with efficient biofilm-targeting agents. The *in vitro* antifungal tolerance of *A. fumigatus* in biofilm has already been reported (Mowat et al., 2007; Seidler et al., 2008; Bugli et al., 2013; Luo et al., 2018). Mowat et al. (2007) showed that amphotericin B, itraconazole, voriconazole, and caspofungin were 1,000 times less efficient on biofilm-life form than on planktonic form. Several studies showed that levofloxacin could be an alternative to treat *S. maltophilia* infections (King et al., 2010; Wu et al., 2013; Herrera-Heredia et al., 2017; Pompilio et al., 2020). This molecule could reduce biofilm biomass (Di Bonaventura et al., 2004; Passerini de Rossi et al., 2009), but some levofloxacin-resistant strains emerge (Wang et al., 2020). Another study suggested the combination of old alternatives, such as rifampicin, with newer agents for critically ill patients infected with *S. maltophilia* (Savini et al., 2010). Rifampicin is known for its anti-biofilm activity and synergistic effect with several antibiotics targeting Gram-positive bacteria (Tang et al., 2013; Yan et al., 2018).

Regarding microbial intra-kingdom interactions, the antimicrobial susceptibility of different bacterial species growing inside polymicrobial biofilms has been investigated (Pompilio et al., 2015; Cendra et al., 2019; Rodríguez-Sevilla et al., 2019). Cross-kingdom interactions impact on antimicrobial susceptibility is still poorly studied, but some authors have described the antimicrobial susceptibility of *Candida albicans* yeast in polymicrobial biofilm with *Staphylococcus aureus* (Harriott and Noverr, 2009; Kong et al., 2016; Rogiers et al., 2018) or *Cutibacterium acnes* (Bernard et al., 2018). To our knowledge, only (Manavathu et al., 2014; Manavathu and Vazquez, 2015) described the antimicrobial susceptibility of an *in vitro* filamentous fungal (*A. fumigatus*) and bacterial (*Pseudomonas aeruginosa*) biofilm. In that model, *A. fumigatus* had the same antifungal susceptibility in mono- and polymicrobial biofilms. Regarding the susceptibility of *P. aeruginosa*, cefepime and imipenem were significantly less efficient in the polymicrobial biofilm than in the monomicrobial biofilm.

We previously showed that *S. maltophilia* inhibited *A. fumigatus* growth and modified hyphae development in a polymicrobial biofilm (Melloul et al., 2016), with strain-dependent manner (Melloul et al., 2018). The aim of the present study was to investigate the *in vitro* antimicrobial response of *A. fumigatus* and *S. maltophilia* in our polymicrobial biofilm in comparison with monomicrobial biofilm.

## Materials and Methods

### Strains and Standardization of Inocula

*Aspergillus fumigatus* (Af) ATCC 13073-GFP (Wasylnka and Moore, 2002) expressing a constitutive Green Fluorescent Protein (AF_REF), and *S. maltophilia* (Sm) ATCC 13637 (SM_REF) were the clinical reference strains used in this study. Two other clinical strains obtained from sputa of CF patients, named AF_CF and SM_CF, were used. AF_CF is *A. fumigatus sensu stricto* as identified by molecular technique using sequence analysis of beta-tubulin gene as previously described (Loeffert et al., 2017). The genomic phylogeny of Sm was recently updated (Vinuesa et al., 2018) and *S. maltophilia* complex was defined, including Sm *sensu stricto* and several related genospecies. SM_CF is *S. maltophilia sensu stricto* through whole genome sequencing and phylogenomic analysis (Mercier-Darty et al., 2020). Both SM_REF and SM_CF belong to genogroup 6 (Hauben et al., 1999; Mercier-Darty et al., 2020), and no major difference in resistance genes was found. Af strains were cultured on 2% malt agar containing 0.05% chloramphenicol at 37°C for 5 days. The fungal suspensions were prepared as previously described to obtain an inoculum of 10^5^ conidia/mL in 3-(N-morpholino) propanesulfonic acid (MOPS) - buffered RPMI 1640 [pH 7.0] with 2% glucose (G) + 10 % fetal bovine serum (FBS) (Sigma-Aldrich, France) (Melloul et al., 2016). Sm strains were streaked out on Luria-Bertani (LB) agar plate at 37°C for 24 h. The bacterial suspensions were also prepared as previously reported to obtain an inoculum of 10^6^ bacteria/mL (Melloul et al., 2016). These inocula were used to test antimicrobial susceptibilities of planktonic cells and biofilms.

### Fungal, Bacterial, and Polymicrobial Biofilm Formation

The *in vitro* biofilm formation in the 96-well plates (Thermo Fisher Scientific Inc, France) was adapted from the protocol previously described (Melloul et al., 2016). Briefly, 50 μL of the fungal (10^5^ conidia/mL) or bacterial (10^6^ bacteria/mL) inoculum was added to 50 μL of MOPS–RPMI (2 % G) + 10 % FBS to form Af or Sm monomicrobial biofilm. The Af-Sm polymicrobial biofilm was produced by simultaneous inoculation of 50 μL of each inoculum per well. The tested microbial associations were AF_REF + SM_REF and AF_CF + SM_CF. Plates were incubated at 37°C in static condition for 24 h to obtain mature biofilms (biofilm-embedded cells), then washed twice with PBS to remove planktonic cells.

### Antimicrobial Agents

Pure antimicrobial powders were obtained from Sigma-Aldrich, France. The antifungal stock solutions of amphotericin B (AMB), itraconazole (ITC), and voriconazole (VRC) were prepared at 10 mg/mL in dimethylsulfoxide (DMSO). The antibiotic stock solutions of levofloxacin (LVX) and rifampicin (RFN) were prepared at 6.4 mg/mL in sterile distilled water and DMSO, respectively. Stock solutions were kept at −20°C until used. Working solutions were then adjusted in MOPS–RPMI (2% G).

### Determination of Minimum Inhibitory Concentration (MIC) and Minimum Bactericidal Concentration (MBC) of Planktonic Cells

**Figure 1:**
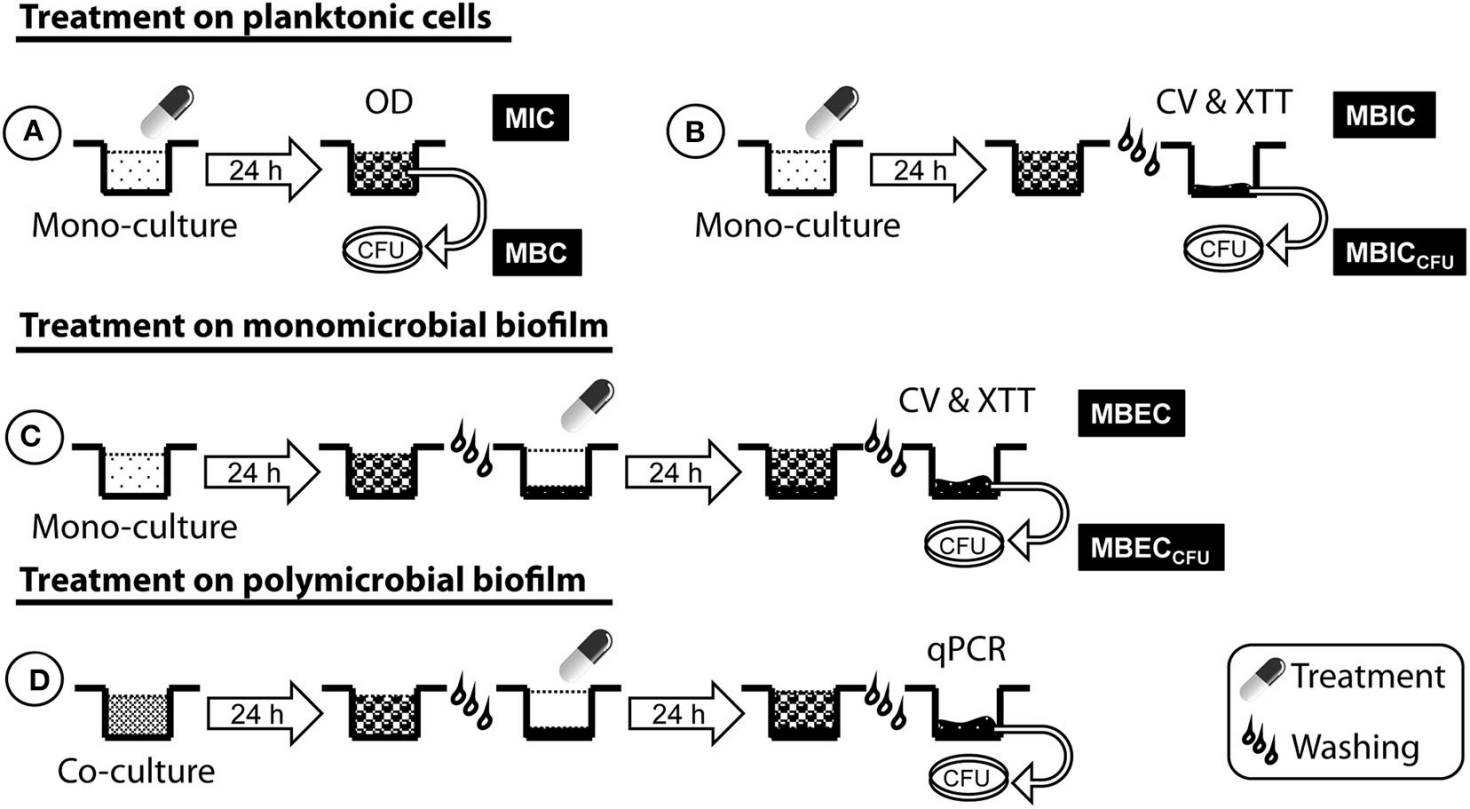
Antimicrobial susceptibility testing of planktonic cells and biofilm forms. **(A,B)** Mono-cultures with conidia or bacteria were simultaneously inoculated with antimicrobial agents for 24 h. Then, the optical density (OD) was measured to obtain the MIC (in planktonic cells), and the wells were washed before crystal violet (CV) and XTT analyses to determine the drug concentration that inhibited the biofilm formation (MBIC). **(C,D)** Mono- and co-cultures were incubated for 24 h to obtain mature mono- and polymicrobial biofilms, and then treated with antimicrobial agents for another 24 h. **(C)** CV and XTT analyses on monomicrobial biofilm enabled determining the drug concentration that eradicated the mature biofilm (MBEC). The viability of Sm following antibacterial treatment was assessed on planktonic cells (MBC), adherent cells of biofilm in formation (MBIC_CFU_), and mature biofilm (MBEC_CFU_). **(D)** Antimicrobial susceptibility of mono- and polymicrobial biofilms was compared using qPCR and viable plate count.

The MICs of AMB, ITC, and VRC were determined by the EUCAST reference microdilution broth technique (Arendrup et al., 2020). The MICs of LVX and RFN were determined following the recommendations from the International Standards Organization (ISO, 2019). MIC was defined as the lowest concentration of drug required for complete growth inhibition with a visual endpoint for Af and a spectrophotometric endpoint for Sm using a microplate reader set at 550 nm (Multiskan FC®, Thermo Fisher Scientific Inc, France). To further compare planktonic and biofilm-embedded cells susceptibilities, the culture conditions of planktonic cells were adjusted to 10^5^ conidia/mL or 10^6^ bacteria/mL and were prepared in MOPS–RPMI (2% G) + 10% FBS medium. In such conditions, the minimum inhibitory concentration (MIC_b_) was determined after 24 h of culture using same endpoints as above (Figure 1A).

MBC was evaluated and defined as the lowest concentration of antibiotic required to reduce Sm CFU by 99.9% as compared with the initial inoculum. MBC was determined by plating 200 μL from each well that showed no visible growth on cation-adjusted Mueller-Hinton (CAMH) (Sigma-Aldrich, France) agar plates incubated at 37°C for 48 h, and CFUs were enumerated. Each experiment was performed in triplicate.

### Determination of Minimum Biofilm Inhibitory Concentration (MBIC) and Viability of *S. maltophilia* Cells Extracted From Biofilm in Formation

To determine the effects of antimicrobial agents on Af and Sm biofilm formation, 50 μL of the fungal (10^5^ conidia/mL) or bacterial (10^6^ bacteria/mL) inoculum was mixed with 50 μL of the antimicrobial agent (2X final concentration) into wells of the 96-well plates and incubated at 37°C in static condition for 24 h. Then, the supernatant was removed, and plates were washed twice with PBS. The final concentration range of the antimicrobial agent was 0.06–8 μg/mL. The lowest concentration of drug required to inhibit at least 90% of the biofilm formation (MBIC) was assessed by the crystal violet (CV) staining method (biomass measurement), and by the 2,3-bis(2-methoxy-4-nitro-5-sulfo-phenyl)-2H-tetrazolium-5carboxanilide (XTT) reduction method (metabolic activity measurement) (Figure 1B). Wells were stained with 200 μL of CV (0.02% for Af, 0.1% for Sm) for 30 min at room temperature, then washed thrice with PBS before adding 200 μL of 30% acetic acid for 10 min. The XTT reduction method was used according to Pierce et al. (2008) with minor modifications. Briefly, a final solution containing 0.5 mg/mL of XTT (Invitrogen, France) + 50 μM (Af) or 10 μM (Sm) of menadione (Merck, Germany) was added into wells and plates were incubated in the dark at 37°C for 2 h. The optical density values of blank wells were subtracted from the test wells. Each test was run in triplicates and three independent experiments were performed.

The viability of adherent bacterial cells was checked on agar plates. The MBIC based on CFU enumeration (MBIC_CFU_) was defined as the lowest concentration of antibiotic required to reduce Sm cell number by 90% on biofilm in formation. Following antibiotic exposure of Sm inoculum, wells were washed, and adherent cells were scraped with swab and plated on LB agar for 24 h at 37°C. All assays were performed in triplicate and repeated three times.

### Determination of Minimum Biofilm Eradication Concentration (MBEC) and Viability of *S. maltophilia* Cells Extracted From Mature Biofilm

Monomicrobial (Af or Sm) mature biofilms were exposed to a range of concentrations of drugs (100 μL) at 37°C for 24 h. The highest concentrations were 256 μg/mL for antifungal and 32 μg/mL for antibacterial agents. For each experiment, some biofilms were not treated with drugs (untreated biofilms). Following washing, the susceptibility testing was performed using CV and XTT (Figure 1C) as described above. Both methods helped determine the MBEC, which was defined as the lowest concentration of drug required to eradicate at least 90% of mature biofilm.

The MBEC based on CFU enumeration (MBEC_CFU_) was evaluated and defined as the lowest concentration of antibiotic required to reduce Sm cell number by 90% on mature biofilm. Following antibiotic treatment of Sm biofilm, wells were washed, and adherent cells were scraped with swab and plated on LB agar for 24 h at 37°C. All assays were performed in triplicate and repeated three times.

### Determination of Polymicrobial Biofilm Susceptibility by Quantitative PCR

Polymicrobial (Af-Sm) mature biofilms were exposed to a range of concentrations of AMB or LVX at 37°C for 24 h. Two-drug combination experiments were performed with AMB at 64 μg/mL and LVX or RFN at 4 μg/mL. The quantity of Af and Sm DNAs was investigated in the drug-treated or drug-free biofilms by qPCR (Figure 1D). Following washing, biofilms were frozen at −20°C for 24 h. After thawing, biofilms were covered with 250 μL of tissue lysis buffer (ATL, Qiagen GmbH, Germany) and prepared as previously described (Melloul et al., 2016). DNA extraction using QIAamp DNA Mini Kit (Qiagen GmbH, Germany) was performed and the qPCR test was conducted following the protocol previously described (Melloul et al., 2016). Data were analyzed using LightCycler software V3.5 and results were expressed in conidial equivalent (CE) or bacterial equivalent (BE) in comparison with a standard curve plotted on DNA samples extracted from co-inoculated solutions with different concentrations of conidia (1–10^8^ conidia) and bacteria (10–10^9^ bacteria). Results obtained from treated biofilms were expressed in percentage of biomass inhibition compared with untreated biofilms. Each testing condition was performed in duplicate for three independent experiments.

### Effect of *A. fumigatus* on *S. maltophilia* Biofilm Susceptibility

#### Comparative Analysis of Viable Bacterial Counts in Monomicrobial and Polymicrobial Biofilms

The effect of Af biomass on the susceptibility of Sm was assessed by a comparative analysis of viable bacterial counts extracted from mono- and polymicrobial biofilms (Figure 1D). One strain association (AF_REF + SM_REF) was used, and the response of SM_REF to LVX (1, 4, and 32 μg/mL) or AMB (64 μg/mL) + LVX (4 μg/mL) was assessed. Biofilms were thoroughly scraped with 200 μL of PBS and collected into tubes, and that was repeated twice for vigorous agitation using MagNA Lyser Instrument (Roche, France). Serial 10-fold dilutions up to 10^−5^ in PBS were performed and 100 μL from each dilution was plated on LB agar supplemented with 16 μg/mL of ITC to prevent Af growth. The number of CFUs was determined after 24 h of incubation at 37°C. Results were expressed in percentage of survival compared with untreated biofilms. All assays were repeated three times for three independent experiments.

#### Effect of Fungal Matrix Degradation on *S. maltophilia* Biofilm Susceptibility

To investigate the effect of AF_REF on the response of SM_REF to LVX, bacterial viability was assessed after enzymatic pretreatment intended to degrade the fungal biofilm extracellular matrix (ECM). The enzymatic degradation protocol was based on a previous research (De Brucker et al., 2015). For such, following Sm and Af-Sm biofilm formation, samples were washed and covered with MOPS–RPMI (2% G) containing 50 μg/mL proteinase K (Qiagen GmbH, Germany) for 2 h at 37°C or with MOPS–RPMI (2% G) as control. Then, biofilms were exposed to LVX at 1 μg/mL for 24 h at 37°C. Bacterial viability was determined using viable plate count as described above. The experiment was performed in triplicate and repeated three times.

### Confocal Laser Scanning Microscopy (CLSM) Observations

For microscopic analyses, biofilms were developed on Lab-Tek^TM^ slides (Thermo Fisher Scientific Inc, France) in a damp chamber. After 16 h of incubation, wells were washed with PBS. AF_REF, which expresses GFP, was visualized with FITC filter. AF_CF was visualized after Calcofluor-white staining (Invitrogen, France) using DAPI filter. Phenotypic modifications of Af in polymicrobial biofilm were investigated and compared with Af phenotype in monomicrobial biofilm by CLSM. Images of biofilms were obtained by Zeiss LSM 510 META confocal (Zeiss, Germany).

### Transmission Electron Microscopy (TEM) Observations

Effects of LVX (8 μg/mL) on polymicrobial biofilms were investigated by TEM. Biofilms were prepared as previously described (Melloul et al., 2016). Briefly, biofilms were first fixed with 2.5% glutaraldehyde-cacodylate (pH 6.5) and then with 2% osmium tetroxide buffer. The fixed samples were dehydrated using a graded ethanol series and embedded in EPON resin for at least 72 h. Ultra-fine sections were cut via ultramicrotome (Leica EM UC7), gently collected on grids, and stained with lead-citrate and uranyl-acetate solutions before observation under TEM (JEOL 100 CX II instrument, Japan).

### Data Analyses

Linear regression and Spearman's rank correlation helped determine the relationship between CV and XTT results. Data failed the normality test (Shapiro–Wilk), hence the use of non-parametric tests. Comparisons of responses of mono- and polymicrobial biofilms to antimicrobial concentrations were performed using multiple linear regressions. Pairwise comparisons relied on Wilcoxon test. Statistical analyses were conducted using JMP 14.0 software. *P* ≤ 0.05 were considered statistically significant.

## Results

### Comparison of Colorimetric Methods

**Figure 2:**
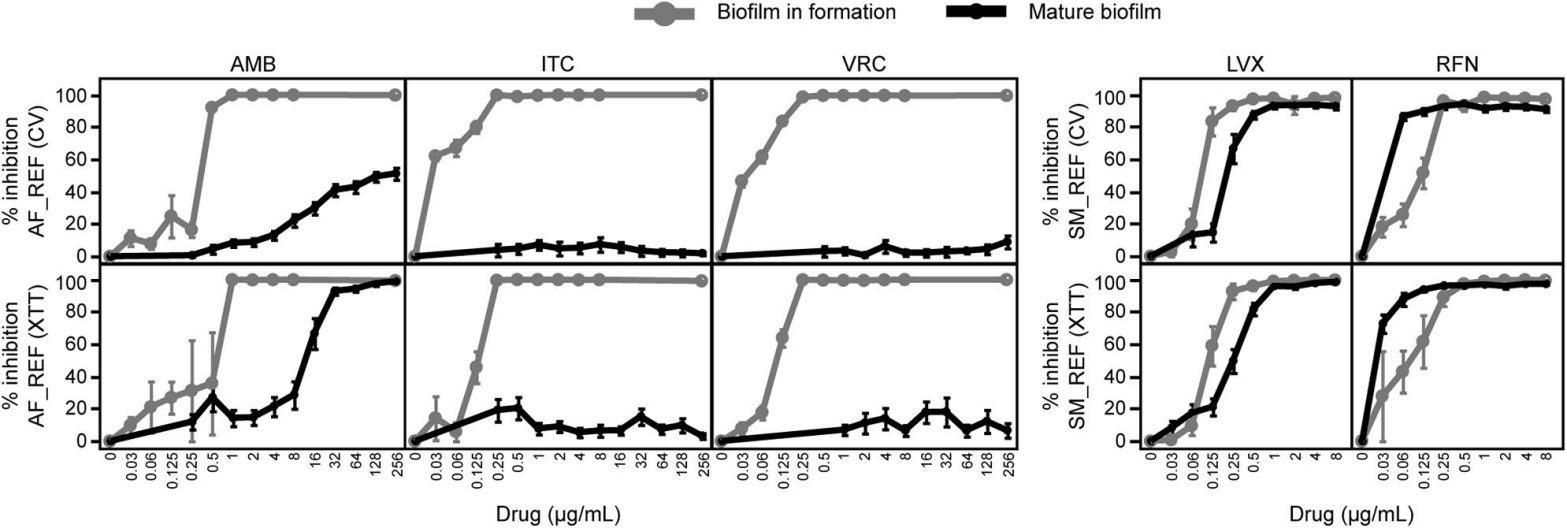
Susceptibilities of biofilms in formation and mature biofilms to drugs. The biomasses and the metabolic activities of biofilms were measured by CV and XTT methods, respectively. Results were expressed in percentages of AF_REF or SM_REF inhibition after antimicrobial treatment compared with untreated controls. AMB, amphotericin B; ITC, itraconazole; VRC, voriconazole; LVX, levofloxacin; RFN, rifampicin.

The effects of various drug concentrations on AF_REF and SM_REF biofilms depending on the used colorimetric method (i.e., CV for biomass measurement or XTT for metabolic activity measurement) are shown in Figure 2. The linear regression and Spearman's rank correlation showed that percentages of inhibition evaluated by CV and XTT methods gave consistent results for antimicrobial susceptibility testing of biofilms in formation (*R*^2^ = 0.83, *p* < 0.0001; Spearman's ρ = 0.8816, *p* < 0.0001) and mature biofilms (*R*^2^ = 0.89, *p* < 0.0001; Spearman's ρ = 0.8562, *p* < 0.0001). Therefore, the effects of antimicrobial agents on the other strains (AF_CF and SM_CF) were evaluated only by the XTT reduction method.

### Effect of Antimicrobial Agents on Planktonic Cells and Biofilm in Formation

MIC_b_ helped to compare planktonic and biofilm-embedded cells susceptibilities. The susceptibility values of planktonic Af and Sm strains are listed in Table 1, which displays the mode for each condition amongst the three performed replicates. MIC_b_ values of the three antifungals were one dilution lower than MIC values, and this was not considered as a major discrepancy. MIC and MIC_b_ of LVX were similar, but showed discrepancy of up to two dilutions for RFN, indicating that Sm strains tended to be more susceptible to RFN in our culture conditions.

**Table 1:** Efficacy of drugs to inhibit growth of planktonic cells and to reduce metabolic activity of biofilm cells.

**Strain**	**Drug**	**MIC**	**MIC_**b**_**	**MBIC_XTT_**	**MBEC_XTT_**
AF_REF	AMB	2	1	1	32
	ITC	0.50	0.25	0.25	>256
	VRC	0.50	0.25	0.25	>256
AF_CF	AMB	2	1	1	8
	ITC	0.50	0.25	0.25	>256
	VRC	0.50	0.25	0.25	>256
SM_REF	LVX	0.125	0.125	0.25	1
	RFN	1	0.25	0.25	0.125
SM_CF	LVX	0.50	0.50	1	2
	RFN	4	2	2	2

*MIC_b_ represented MIC assessed with adapted culture conditions. Concentrations are expressed in μg/mL. AMB, amphotericin B; ITC, itraconazole; VRC, voriconazole; LVX, levofloxacin; RFN, rifampicin; MIC, minimum inhibitory concentration; MBIC, minimum biofilm inhibitory concentration based on XTT readout; MBEC, minimum biofilm eradication concentration based on XTT readout*.

AF_REF and AF_CF were equally susceptible to AMB, ITC, and VRC (MIC_b_s 1, 0.25, and 0.25 μg/mL, respectively). SM_REF and SM_CF were both susceptible to LVX (MIC_b_s ≤ 0.50 μg/mL), and MIC_b_ of RFN for SM_CF (2 μg/mL) was 8-fold higher than that for SM_REF (0.25 μg/mL).

Overall, the susceptibility of planktonic cells (MIC_b_) measured by turbidity was similar to the susceptibility of biofilm in formation (MBIC_XTT_) measured by XTT (Table 1).

### Decrease of Antimicrobial Susceptibility on Monomicrobial Mature Biofilm Compared With Planktonic Cells and Biofilm in Formation

Antifungal and antibacterial agents exhibited concentration-dependent activities against AF_REF and SM_REF biofilms (Figure 2). The lowest drug concentrations required to reduce metabolic activity of biofilm in formation (MBIC_XTT_) and mature biofilm (MBEC_XTT_) are shown in Table 1. AMB exhibited a greater effect on biofilm formation of Af since MBIC_XTT_s (1 μg/mL) were 8 and 32-fold lower than MBEC_XTT_s for AF_CF and AF_REF, respectively. ITC and VRC were efficient to inhibit biofilm formation of both Af strains (MBIC_XTT_s = 0.25 μg/mL), but not to eradicate mature biofilms (MBEC_XTT_s > 256 μg/mL). Only AMB was efficient on mature biofilms with a slightly greater effect on AF_CF (MBEC_XTT_ = 8 μg/mL) compared with AF_REF (MBEC_XTT_ = 32 μg/mL). The MBIC_XTT_s of LVX (0.25 and 1 μg/mL for SM_REF and SM_CF, respectively) were lower than MBEC_XTT_s (1 and 2 μg/mL). LVX exhibited a slightly greater effect to reduce metabolic activity of Sm biofilms in formation than Sm mature biofilms. For RFN, MBIC_XTT_s, and MBEC_XTT_s were similar, indicating that RFN had a similar effect on biofilm formation and mature biofilm according to the XTT results.

The viability of antibiotic-treated Sm cells in planktonic cultures (MBC), biofilm in formation (MBIC_CFU_), and mature biofilm (MBEC_CFU_) is shown in Table 2. The MBC of LVX was similar to its MIC_b_, whereas the MBC of RFN was at least 16-fold higher than MIC_b_ for both Sm strains (Tables 1, 2). LVX reached its bactericidal effect on Sm strains at lower concentrations than RFN.

**Table 2:** Efficacy of drugs to reduce cell numbers on planktonic cultures and biofilm forms of *S. maltophilia*.

**Strain**	**Drug**	**MBC**	**MBIC_**CFU**_**	**MBEC_**CFU**_**
SM_REF	LVX	0.25	0.25	8
	RFN	32	0.25	16
SM_CF	LVX	1	4	32
	RFN	>32	16	>32

*Concentrations are expressed in μg/mL. LVX, levofloxacin; RFN, rifampicin; MBC, minimum bactericidal concentration; MBIC_CFU_, minimum biofilm inhibitory concentration based on CFU enumeration; MBEC_CFU_, minimum biofilm eradication concentration based on CFU enumeration*.

The efficacy of LVX to reduce cell numbers on planktonic cultures (MBC) and biofilm in formation (MBIC_CFU_) was higher than on mature biofilm (MBEC_CFU_), for both Sm strains (Table 2). LVX exhibited a slightly greater effect on SM_REF (MBEC_CFU_ = 8 μg/mL) compared with SM_CF (MBEC_CFU_ = 32 μg/mL). The MBC of RFN was similar to its MBEC_CFU_ for both strains, but the MBEC_CFU_ was up to 64-fold higher than MBIC_CFU_. Thus, RFN only seems to reduce the number of adherent Sm cell by inhibiting the biofilm formation.

### Fungal Growth Inhibition and Fungal Phenotype Modification in the Presence of *S. maltophilia*

**Figure 3:**
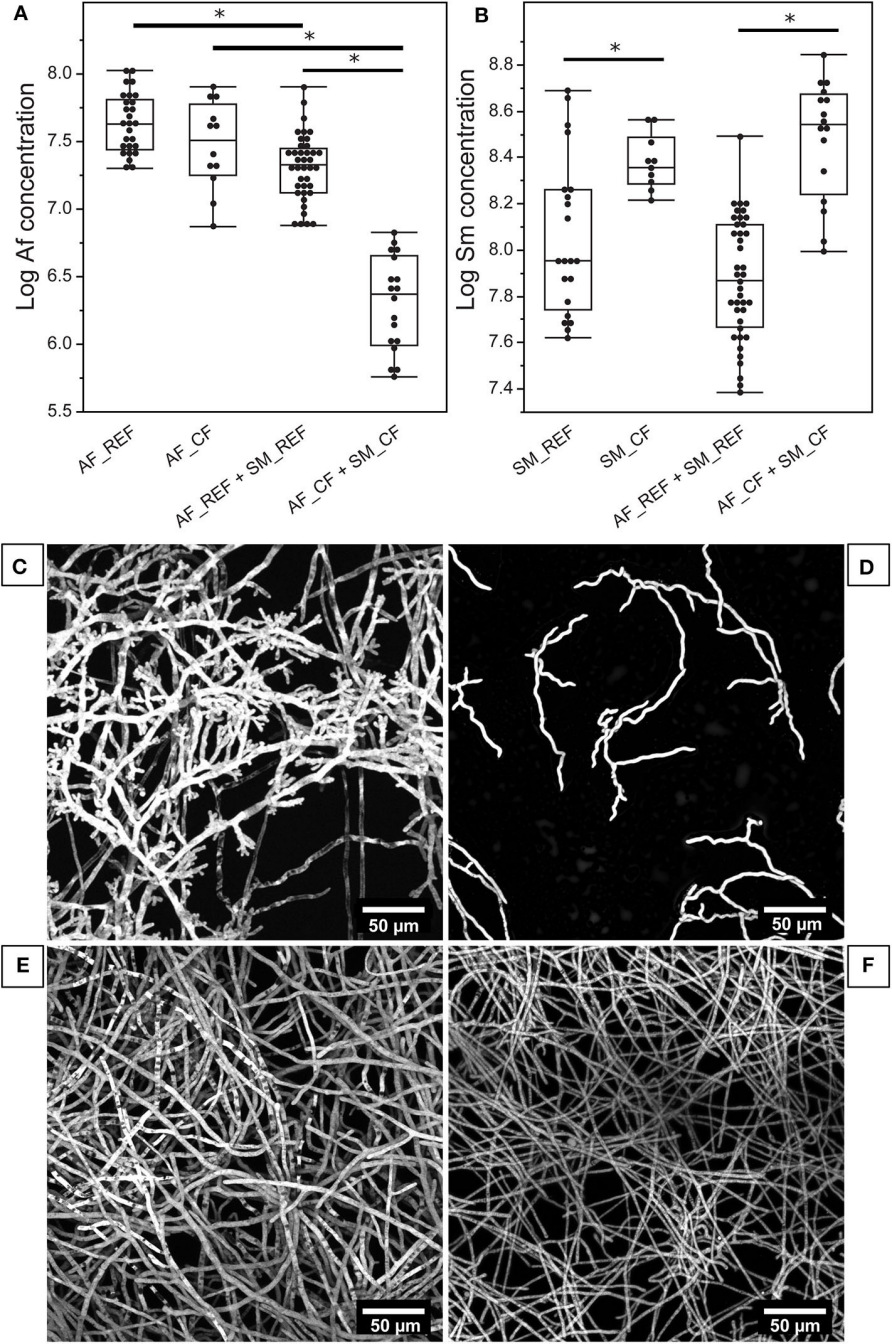
Quantification of fungal and bacterial concentrations in biofilms and phenotype modifications of *A. fumigatus* in the presence of *S. maltophilia*. **(A,B)** Assessment of Af or Sm growth in mono- and polymicrobial biofilms after 24 h of culture by qPCR. *p < 0.05. **(C)** AF_REF phenotype in polymicrobial biofilm with SM_REF. **(D)** AF_CF phenotype in polymicrobial biofilm with SM_CF. **(E)** AF_REF phenotype in monomicrobial biofilm. **(F)** AF_CF phenotype in monomicrobial biofilm. Af, *A. fumigatus*; Sm, *S. maltophilia*.

In AF_REF + SM_REF biofilm, the fungal growth was significantly reduced (*p* < 0.0001) but not the bacterial growth (*p* = 0.0520), in comparison with the corresponding monomicrobial biofilms (Figures 3A,B). We obtained the same trend for CF strains: AF_CF in mono- vs. polymicrobial biofilm (*p* < 0.0001) and SM_CF in mono- vs. polymicrobial biofilm (*p* = 0.1706; Figures 3A,B). The fungal growth inhibition was higher for the association of CF strains than for REF strains. Specifically, the growth of AF_REF and AF_CF in polymicrobial biofilms was respectively reduced by 2 and 10 compared with that in fungal biofilms. This difference can be attributed to the difference of Sm fitness (Figure 3B). SM_CF grew significantly faster than SM_REF in polymicrobial biofilm (*p* < 0.0001) and thus induced a larger inhibition of fungal growth. Moreover, the fungal phenotype was modified and showed highly branched hyphae in the presence of bacteria for both associations (Figures 3C,D) in comparison with the corresponding Af monomicrobial biofilm (Figures 3E,F).

### Modification of *A. fumigatus* Susceptibility to AMB in Polymicrobial Biofilm

**Figure 4:**
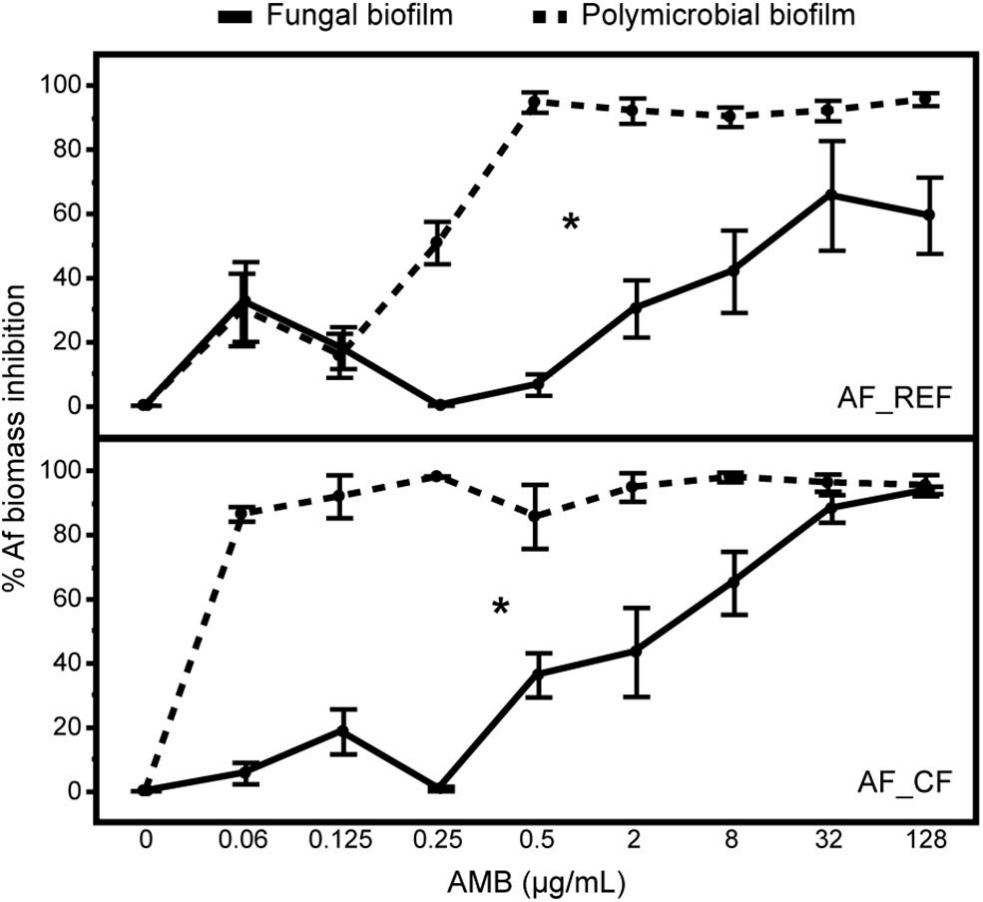
Effects of amphotericin B on *A. fumigatus* in fungal and polymicrobial biofilms. Assessment of AMB activity on AF_REF or AF_CF in fungal (solid line) and polymicrobial (broken line) biofilms by qPCR. Percentages of biomass inhibition were calculated based on untreated biofilms results. *p < 0.05; Af, *A. fumigatus*; AMB, amphotericin B.

Since no ITC and VRC activities on Af monomicrobial biofilms were found (Table 1), we focused on AMB activity for the following experiments. Af susceptibility to AMB in mono- and polymicrobial biofilms was tested for both strain associations: AF_REF + SM_REF and AF_CF + SM_CF. The percentage of fungal biomass inhibition measured in AMB-treated biofilms was significantly higher in polymicrobial biofilms for both Af strains (multiple linear regressions, biofilm ^*^ AMB concentration effect: *p* < 0.001; Figure 4); i.e., the fungus was more susceptible to AMB in the presence of Sm. The AMB concentrations required to obtain 90% of AF_REF or AF_CF biomass inhibition were at least 32 μg/mL in fungal biofilm and 0.5 or ≤ 0.06 μg/mL in polymicrobial biofilm. The difference in Af susceptibility between fungal and polymicrobial biofilms was observed from 0.25 and ≤ 0.06 μg/mL of AMB for AF_REF and AF_CF, respectively. Overall, Af susceptibility to AMB in polymicrobial biofilm was at least 64-fold higher compared with fungal biofilm (Figure 4), whereas the fungal growth was reduced by 2 times with SM_REF and 10 times with SM_CF (Figure 3A).

### Modification of *S. maltophilia* Susceptibility to LVX in Polymicrobial Biofilm

**Figure 5:**
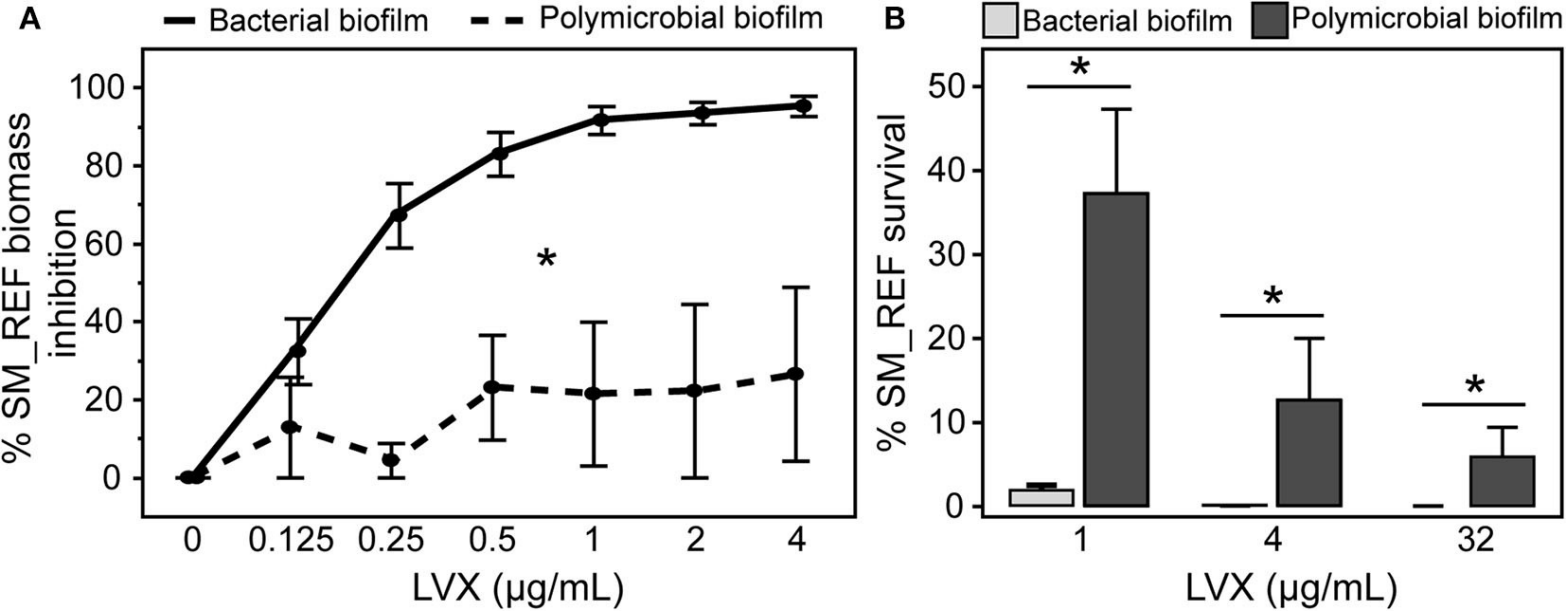
Effects of levofloxacin on SM_REF in bacterial and polymicrobial biofilms. **(A)** Assessment of LVX activity on SM_REF in bacterial (solid line) and polymicrobial (broken line) biofilms by qPCR. **(B)** Percentages of SM_REF survival following LVX (1, 4, and 32 μg/mL) treatment in bacterial (light gray) and polymicrobial (dark gray) biofilms using viable plate count. *p < 0.05; LVX, levofloxacin.

**Figure 6:**
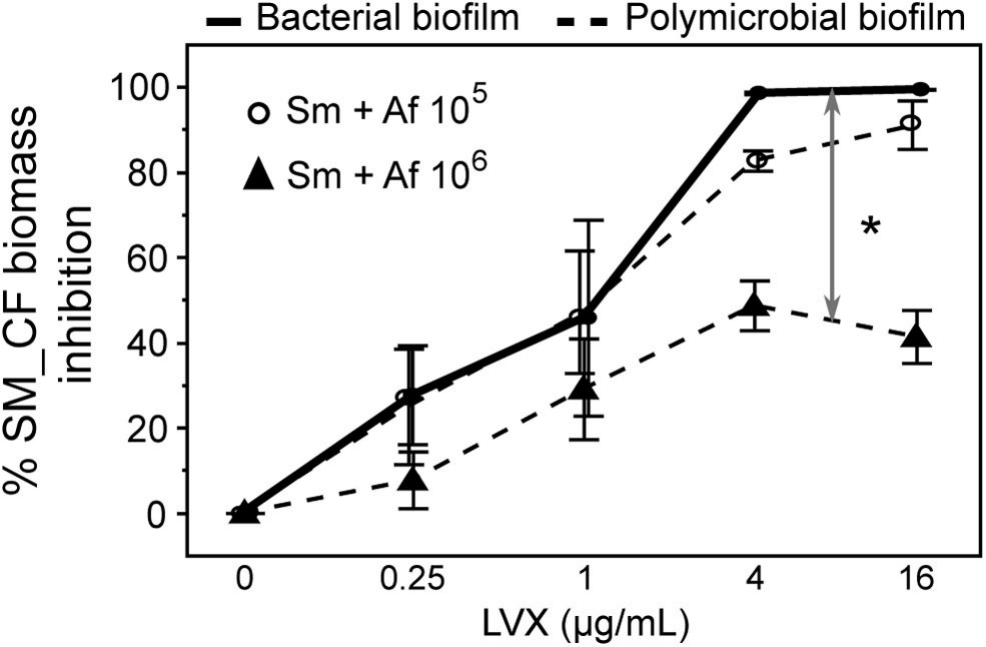
Effects of levofloxacin on SM_CF in bacterial and polymicrobial biofilms. Susceptibility of SM_CF to LVX was assessed in bacterial (solid line) and polymicrobial biofilms (broken lines) with an initial AF_CF inoculum of 10^5^ conidia/mL (circle) or 10^6^ conidia/mL (triangle) by qPCR. *p < 0.05; LVX, levofloxacin.

**Figure 7:**
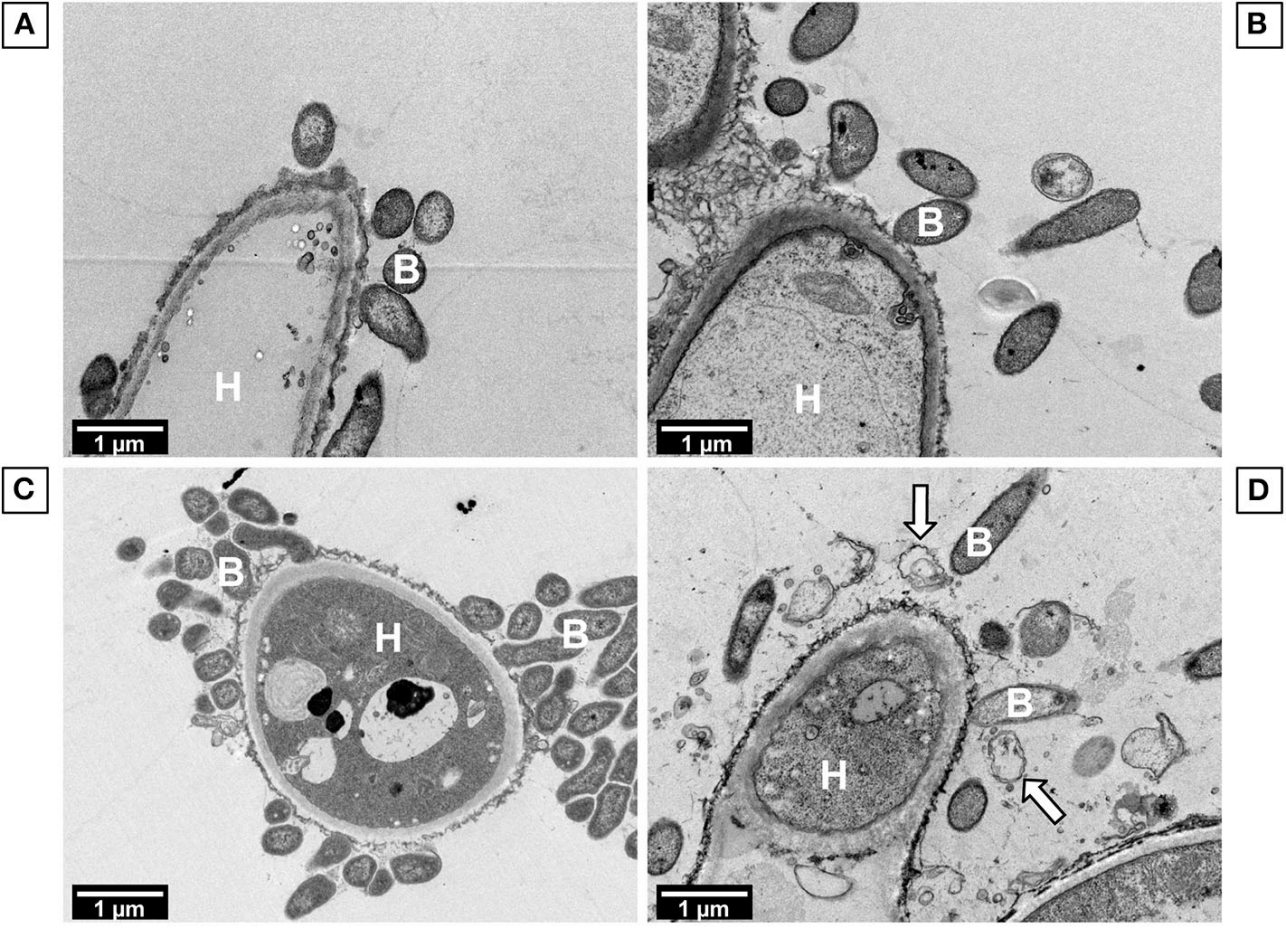
TEM observations of *A. fumigatus*-*S. maltophilia* biofilms exposed to levofloxacin. **(A)** AF_REF + SM_REF untreated biofilm. **(B)** AF_REF + SM_REF biofilm treated with 8 μg/mL LVX. **(C)** AF_CF + SM_CF untreated biofilm. **(D)** AF_CF + SM_CF biofilm treated with 8 μg/mL LVX. H, hypha; B, bacteria. The white arrows show damages to bacterial cells.

The susceptibility of Sm in bacterial and polymicrobial biofilms was compared in response to LVX. Figure 5A results showed that LVX had a greater effect on SM_REF in bacterial biofilm than in polymicrobial biofilm (multiple linear regressions, *p* < 0.0001). In the bacterial biofilm, the inhibition level raised gradually and proportionally with the increase in LVX concentration until 90% at 1 μg/mL. This result was consistent with that of MBEC_XTT_ (1 μg/mL, Table 1). Concerning SM_REF in polymicrobial biofilm, LVX had a limited effect (20% inhibition, regardless of LVX concentration) (Figure 5A). In addition to these results, the bacterial survival was assessed by subculturing the LVX-treated biofilms (Figure 5B). The results showed a significantly higher survival rate of SM_REF in polymicrobial biofilm than in bacterial biofilm following LVX treatment at 1 μg/mL (*p* = 0.0003), 4 μg/mL (*p* = 0.0002), and 32 μg/mL (*p* = 0.0025). In the bacterial biofilm, 2% (~10^6^ bacteria/mL) and 0.001% (10^3^ bacteria/mL) of survival rates were recorded following exposure to 1 and 32 μg/mL of LVX, respectively. In contrast, in the polymicrobial biofilm, almost 40 and 6% of Sm were still alive after exposure to 1 and 32 μg/mL of LVX, respectively. For SM_CF, qPCR results showed a similar antibacterial effect of LVX on the bacterial and polymicrobial biofilms (multiple linear regressions, *p* = 0.8550; Figure 6). Regardless of the LVX concentration used, the presence of AF_CF had no effect on SM_CF susceptibility to LVX, while the presence of AF_REF decreased the susceptibility of SM_REF to LVX. This difference was probably due to the higher inhibition of Af growth exhibited in the association of CF strains (Figure 3A). In addition, TEM experiments were performed to visualize effects of LVX (8 μg/mL) on polymicrobial biofilms. SM_REF cells grown with AF_REF did not show any signs of severe damage after LVX treatment (Figures 7A,B), whereas SM_CF cells appeared broken and emptied of their contents in LVX-treated polymicrobial biofilm (Figures 7C,D).

### Decrease of *S. maltophilia* Susceptibility to LVX in Polymicrobial Biofilm Is Related to the *A. fumigatus* Biomass

Since the fungal biomass in the AF_CF + SM_CF biofilm was lower than in the AF_REF + SM_REF biofilm (Figure 3A), we hypothesized that the fungal biomass was responsible for the significant decrease of SM_REF susceptibility to LVX observed in polymicrobial biofilm (Figure 5A), in contrast to SM_CF (Figure 6). To test this hypothesis, we carried out experiments using different concentrations of AF_CF (10^5^ or 10^6^ conidia/mL) for the same SM_CF concentration (10^6^ bacteria/mL) in order to reduce the fungal growth inhibition caused by the bacteria. There was a bigger fungal biomass (+ 0.5 log CE/mL, data not shown) in the polymicrobial biofilm formed with 10^6^ conidia/mL than that observed in the polymicrobial biofilm with 10^5^ conidia/mL. In the bacterial biofilm, 4 μg/mL of LVX was enough to achieve 90% SM_CF inhibition, which was not the case in the polymicrobial biofilm (performed with 10^6^ conidia/mL) where 16 μg/mL of LVX could not exceed 50% inhibition. The presence of higher AF_CF biomass helped to protect SM_CF from LVX (multiple linear regressions, *p* = 0.0121; Figure 6).

### Role of the Fungal ECM in *S. maltophilia* Protection From LVX

**Figure 8:**
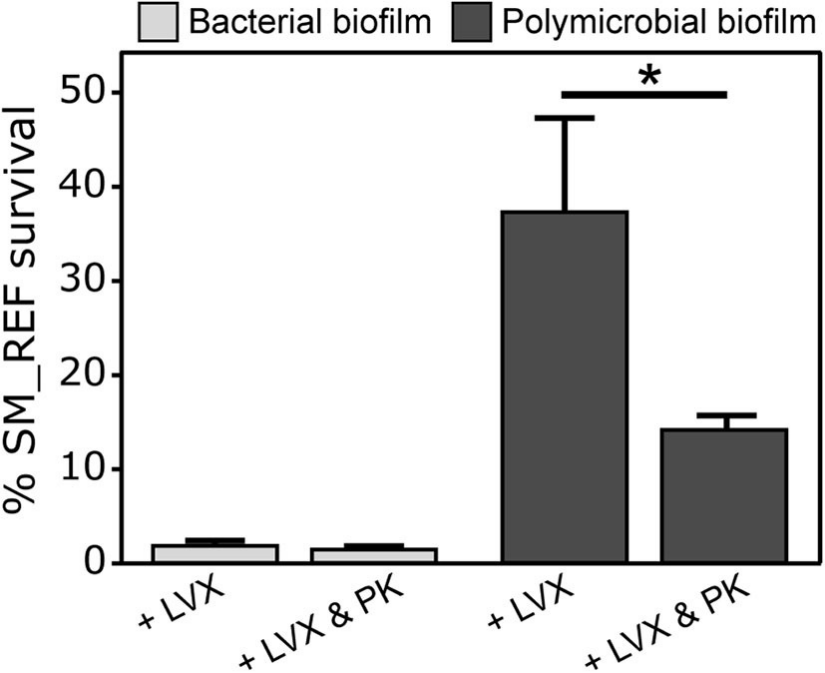
Effects of *A. fumigatus* matrix on *S. maltophilia* biofilm susceptibility to levofloxacin. Bacterial and polymicrobial biofilms were treated with or without proteinase K (50 μg/mL) for 2 h before LVX treatment at 1 μg/mL. Percentages of SM_REF survival was assessed using viable plate count. *p < 0.05; LVX, levofloxacin; PK, proteinase K.

Fungal ECM may have a role in decreasing Sm susceptibility to LVX in polymicrobial biofilm. To support this hypothesis, Sm and Af-Sm biofilms were pretreated with proteinase K to degrade proteins of ECM before analyzing the bacterial survival following LVX treatment at 1 μg/mL. Proteinase K did not affect the SM_REF response to LVX in bacterial biofilm (*p* = 0.8563; Figure 8). However, a significant decrease of SM_REF survival following LVX treatment was observed in polymicrobial biofilm pretreated with Proteinase K compared with unpretreated biofilm (*p* = 0.0333; Figure 8), suggesting that AF_REF ECM is involved in the protection of SM_REF from LVX.

### Antifungal and Antibacterial Combination Strategies to Treat Polymicrobial Biofilm

Antifungal-antibacterial combinations were tested on Af-Sm biofilm in order to impair both pathogens and to provide more evidence that Af plays a role in protecting Sm from the effects of antibiotics. Bacterial and polymicrobial biofilms of REF strains were exposed to AMB at 64 μg/mL combined with LVX or RFN at 4 μg/mL, and to each drug alone.

In polymicrobial biofilm, the susceptibility of AF_REF to AMB alone or in combination with antibiotics did not differ (AMB vs. AMB + LVX: *p* = 0.2027; AMB vs. AMB + RFN: *p* = 0.3801) (data not shown). These results suggested that the antibiotics did not affect the antifungal efficacy of AMB on Af in polymicrobial biofilm.

**Figure 9:**
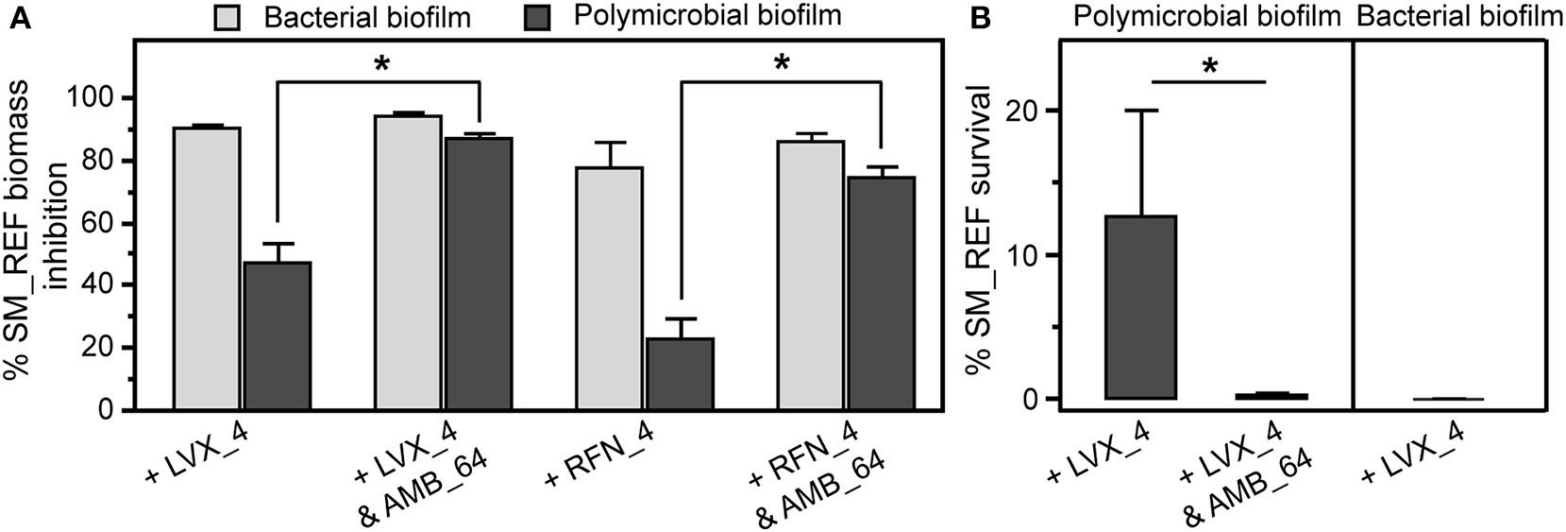
Effects of antifungal-antibacterial combination on *A. fumigatus-S. maltophilia* biofilm. **(A)** Susceptibility of SM_REF in bacterial (light gray) and polymicrobial (dark gray) biofilms to LVX and RFN alone or in two-drug combination with AMB assessed by qPCR. **(B)** Percentages of SM_REF survival following LVX alone or in two-drug combination with AMB treatment in bacterial and polymicrobial biofilms using viable plate count. *p < 0.05; AMB_64, amphotericin B (64 μg/mL); LVX_4, levofloxacin (4 μg/mL); RFN_4, rifampicin (4 μg/mL).

When the polymicrobial biofilm was treated with AMB (64 μg/mL), the growth of SM_REF was significantly increased compared with untreated biofilms (*p* < 0.0001, data not shown). SM_REF biomass was ~5 × 10^7^ BE/mL in the untreated polymicrobial biofilm vs. 3 × 10^8^ BE/mL in the AMB-treated. For such, the results of the two-drug combination were expressed in percentages of growth inhibition and survival compared with AMB-treated biofilm. The qPCR analysis demonstrated that AMB in combination with LVX or RFN significantly improved the antibacterial effect against Sm in polymicrobial biofilm in comparison with LVX or RFN alone (LVX vs. AMB + LVX: *p* < 0.0001; RFN vs. AMB + RFN: *p* < 0.0001; Figure 9A). Remarkably, the susceptibility of Sm in polymicrobial biofilm to antifungal-antibacterial combination was not significantly different from the susceptibility of Sm in bacterial biofilm to each antibacterial alone (Sm-LVX vs. Af-Sm-AMB + LVX: *p* = 0.8668; Sm-RFN vs. Af-Sm-AMB + RFN: *p* = 0.3963; Figure 9A). AMB + LVX combination was significantly more efficient against Sm in polymicrobial biofilm (90% of Sm growth inhibition) than AMB + RFN (75% of Sm growth inhibition; *p* = 0.0002). Therefore, survival experiments were performed with AMB in combination with LVX (Figure 9B). A higher reduction of Sm survival in polymicrobial biofilm following AMB + LVX treatment compared with LVX alone (*p* = 0.0006) was obtained by viable plate count. Moreover, AMB + LVX combination against Sm in polymicrobial biofilm was as efficient as LVX alone against Sm in bacterial biofilm (*p* = 0.1096; Figure 9B). These results suggest that the inhibition of Af with AMB prompted Sm susceptibility to antibiotics.

## Discussion

Managing cross-kingdoms polymicrobial infections, especially of biofilm-forming microbes, remains an outstanding challenge to overcome resistance to a wide range of clinical antimicrobial agents. Conventional treatment of infectious diseases relies on standard susceptibility testing of planktonic cells, which does not take into account microbial interactions. For such, this approach becomes unsuitable against polymicrobial biofilm with sessile cells embedded in ECM. We previously showed *A. fumigatus*-*S. maltophilia* interactions in polymicrobial biofilm (Melloul et al., 2016, 2018), and from there we sought to determine whether the antimicrobial response of both pathogens would be modified in their polymicrobial biofilm.

The present study highlights the modulated antimicrobial response of a filamentous fungal-bacterial biofilm. We showed that *A. fumigatus* monomicrobial biofilms were susceptible to amphotericin B, but not to itraconazole, and voriconazole. Amphotericin B susceptibility of *A. fumigatus* increased when it was embedded in polymicrobial biofilm with *S. maltophilia*. Levofloxacin and rifampicin were efficient in inhibiting *S. maltophilia* monomicrobial biofilms, but much higher concentrations were needed to eradicate it. *S. maltophilia* susceptibility to levofloxacin decreased in the polymicrobial biofilm and was fungal biomass-dependent. The inhibited effect of *S. maltophilia* on *A. fumigatus* growth was more pronounced for the association of CF strains than the reference strains. The combination of amphotericin B with levofloxacin or rifampicin was efficient to impair both pathogens in the polymicrobial biofilm.

Antifungal susceptibility of *A. fumigatus* biofilms was assessed by the XTT reduction method, already used several years ago (Mowat et al., 2007; Seidler et al., 2008). We focused on viability test to assess antibacterial susceptibility of *S. maltophilia* biofilms, which have been used in previous research (Di Bonaventura et al., 2004; Pompilio et al., 2016). The antibiosis effect of *S. maltophilia* on *A. fumigatus* growth was revealed by qPCR. This technique was also used to compare the antimicrobial susceptibility of species in mono- and polymicrobial biofilms.

Our results revealed clear differences in antifungal susceptibilities between biofilms in formation and mature biofilms of *A. fumigatus* (i.e., MBIC_XTT_ vs. MBEC_XTT_, Table 1). The formation of *A. fumigatus* biofilm was prevented by the three antifungal agents (MBIC_XTT_s ≤ 1 μg/mL). *A. fumigatus* mature biofilm was inhibited by amphotericin B (MBEC_XTT_ range = 8–32 μg/mL), but not by the two azoles (MBEC_XTT_s > 256 μg/mL), as already shown in the work of Mowat et al. (2007). These results suggest that azoles could be useful in preventing biofilm formation rather than in treating mature biofilm. To date, the reasons for the decreased susceptibility of *A. fumigatus* biofilm to drugs have not been fully elucidated (Latgé and Chamilos, 2019).

Considering the results with azoles, we focused on amphotericin B activity on *A. fumigatus* in polymicrobial biofilms with *S. maltophilia* and we found an increase susceptibility compared with their corresponding *A. fumigatus* monomicrobial biofilms (Figure 4). A possible explanation is the modification of fungal phenotype due to the presence of bacteria. In our model, the modification of *A. fumigatus* cell wall by *S. maltophilia* (Melloul et al., 2018) resembles the one induced by caspofungin or dirhamnolipids (diRhls) secreted by *P. aeruginosa* which specifically inhibit the fungal 1,3-glucan synthase activity (Briard et al., 2017). We suppose that some diRhls-like molecules secreted by *S. maltophilia* would modify *A. fumigatus* phenotype similarly to echinocandins. This could justify the impairment of *A. fumigatus* response to amphotericin B, as showed for clinical *Aspergillus* spp. strains treated with a combination of amphotericin B and echinocandins (Panackal et al., 2014). Iron could be another important parameter in this interaction between *S. maltophilia* and *A. fumigatus*. The role of two siderophores secreted by *P. aeruginosa* (pyoverdine and pyochelin) involved in the reduction of *A. fumigatus* growth has been analyzed (Sass et al., 2018; Briard et al., 2019) and the production of catecholate siderophores from *S. maltophilia* clinical strains has been shown (García et al., 2012; Nas and Cianciotto, 2017). In our polymicrobial model, we can hypothesize that siderophores secreted by *S. maltophilia* could deprive *A. fumigatus* from iron. In turn, iron deficiency could increase *A. fumigatus* susceptibility, as reported by Zarember et al. (2009) who showed that iron deprivation gave better *A. fumigatus* response to amphotericin B treatment.

The viability of antibiotic-treated *S. maltophilia* in planktonic cultures (MBC), biofilm in formation (MBIC_CFU_), and mature biofilm (MBEC_CFU_) is shown in Table 2. Results showed a reduced levofloxacin susceptibility of biofilm-embedded bacteria. This finding is consistent with the results of previous studies (Passerini de Rossi et al., 2009; Pompilio et al., 2016). The *in vitro* activity of other fluoroquinolones against biofilm-embedded *S. maltophilia* cells has already been reported (Di Bonaventura et al., 2004; Passerini de Rossi et al., 2009; Wu et al., 2013; Wang et al., 2016). However, no study analyzed their effect on *S. maltophilia* in polymicrobial biofilms with a filamentous fungus. Our study demonstrated that *S. maltophilia* response to levofloxacin was impacted by the presence of *A. fumigatus*. Manavathu et al. (2014) documented a decrease of bacterial susceptibility in *A. fumigatus*-*P. aeruginosa* polymicrobial biofilm with no explanation of the underlying mechanism. Our work demonstrated that levofloxacin effect on *S. maltophilia* in polymicrobial biofilms, of both associations of strains, decreased and was fungal biomass-dependent. The network of *A. fumigatus* hyphae could protect *S. maltophilia* from levofloxacin. The higher fitness of SM_CF, in comparison with SM_REF, could account for the larger antibiosis effect of the CF bacterial strain on the CF fungal growth in polymicrobial biofilm. Alike, in our latest study, we showed that *S. maltophilia* antibiosis on *A. fumigatus* was dependent on the bacterial fitness (Melloul et al., 2018). The fungal biomass of AF_CF + SM_CF biofilm was insufficient to protect the bacteria from levofloxacin, but upon increasing the fungal biomass (using a larger initial inoculum), the polymicrobial biofilm provided SM_CF with a better protection from levofloxacin (Figure 6). We further suggested that *A. fumigatus* ECM could prevent drug diffusion by acting as a physical barrier and enhance *S. maltophilia* tolerance to levofloxacin. This hypothesis was partially validated using proteinase K pretreatment to damage the ECM structure of the polymicrobial biofilm, similarly to what De Brucker et al. (2015) did for *C. albicans* and *Escherichia coli* to obtain a lower bacterial tolerance to ofloxacin. Moreover, matrix components released by *A. fumigatus* could alter the physiology of *S. maltophilia* by restricting penetration of nutrients or oxygen into the aggregates. Indeed, Stewart et al. (2015) showed that a low oxygen level seems to be the primary mechanism for tolerance of biofilms to quinolones. Further investigations are warranted to identify the fungal ECM components, which could promote antibacterial protection of *S. maltophilia* in *A. fumigatus*-*S. maltophilia* biofilm.

Some studies put forwards the use of rifampicin to treat *S. maltophilia* infections (Savini et al., 2010; Betts et al., 2014), but to our knowledge, we are the first to explore its activity against *S. maltophilia* biofilm. Our experiments needed high doses of rifampicin to eradicate planktonic cells (MBC, Table 2) and mature biofilms (MBEC_CFU_, Table 2) of both *S. maltophilia* strains. Rifampicin was only efficient to eradicate the SM_REF cells of biofilm in formation (MBIC_CFU_, Table 2).

To date, few studies explored *in vitro* effects of antifungal agents in combination with antibacterial agents against polymicrobial biofilms. We demonstrated that levofloxacin or rifampicin combined with a high dose of amphotericin B was significantly more efficient to eradicate *S. maltophilia* in polymicrobial biofilm than the antibiotic alone (Figure 9). This result corroborates the protective effect of *A. fumigatus* on *S. maltophilia* in polymicrobial biofilm, since once the fungus was destroyed by amphotericin B, the antibiotic had a stronger effect on the bacteria.

Most CF patients with *A. fumigatus* infection are put on azole therapies though their efficacy is discussed (Burgel et al., 2016). In the same way, the success of management of *S. maltophilia* infections in CF patients remains unclear (Amin and Waters, 2016). Finally, treatment failure could be attributed in some cases to the *in vivo* presence of biofilm forms, as it was proposed for *A. fumigatus* (Ramage et al., 2011). Our *in vitro* results demonstrated that the microbial interactions between *A. fumigatus* and *S. maltophilia* mutually modulate their responses to antimicrobial agents. This could be particularly relevant in CF patients where lungs can be characterized by decreased oxygen pressure. Also, these findings add a toll on therapeutic decision-making, since the microbial interactions and the biofilm-forming ability are not usually taken into account to design treatment options.

In conclusion, microbial interactions within polymicrobial biofilms can modulate the antimicrobial response of pathogens. In polymicrobial biofilms, *S. maltophilia* increased the antifungal susceptibility of *A. fumigatus* to amphotericin B, whereas *A. fumigatus* protected *S. maltophilia* from levofloxacin. Further work analyzing the underlying mechanisms of antimicrobial combinations on polymicrobial biofilms would be valuable in the future.

## Data Availability Statement

The raw data supporting the conclusions of this article will be made available by the authors, without undue reservation.

## Author's Note

This work was presented in part at the 6th European Congress on Biofilms (EUROBIOFILMS) (Glasgow, Scotland, September 2019).

## Author Contributions

EM and FB conceived the design of the study. EM supervised the experiments. LR performed the experiments and the statistical analysis. LR and EM designed the figures. LR, EM, and FB participated in results analysis. P-LW and ED helped in the interpretation of antibacterial and antifungal activities, respectively. J-WD and GR analyzed *S. maltophilia* strains. LR, EM, JG, and FB drafted the manuscript. All authors read and approved the final manuscript.

## Conflict of Interest

During the past 5 years, ED has received research grants from Gilead and MSD; travel grants from Gilead, MSD, Pfizer, and Astellas, and speaker's fee from Gilead, MSD, and Astellas. FB has received research grants from MSD; travel grants from Gilead, MSD, Pfizer, and speaker's fee from Gilead, MSD, and Pfizer. The remaining authors declare that the research was conducted in the absence of any commercial or financial relationships that could be construed as a potential conflict of interest. The handling editor declared a past co-authorship with the authors ED and FB.

## Abbreviations

Af: *A. fumigatus*
Sm: *S. maltophilia*
AMB: amphotericin B
ITC: itraconazole
VRC: voriconazole
LVX: levofloxacin
RFN: rifampicin.
